# Photonic quasicrystal of spin angular momentum

**DOI:** 10.1126/sciadv.adv3938

**Published:** 2025-04-30

**Authors:** Min Lin, Xinxin Gou, Zhenwei Xie, Aiping Yang, Luping Du, Xiaocong Yuan

**Affiliations:** ^1^ Nanophotonics Research Centre, Shenzhen Key Laboratory of Micro-Scale Optical Information Technology, Institute of Microscale Optoelectronics and State Key Laboratory of Radio Frequency Heterogeneous Integration, Shenzhen University, Shenzhen 518060, China.; ^2^ Research Institute of Interdisciplinary Science and School of Materials Science and Engineering, Dongguan University of Technology, Dongguan 523808, China.

## Abstract

Quasicrystals, characterized by long-range order without translational symmetry, have catalyzed transformative advances in various fields, including optics in terms of field quasicrystals. To our knowledge, we present the first demonstration of photonic quasicrystals formed by spin angular momentum, unveiling previously unidentified spin-orbit coupling effects absent in traditional field quasicrystals. A de Bruijn tiling like theoretical framework was built elucidating the formation mechanism of spin quasicrystals for diverse symmetries. Moreover, the configurations of these spin textures can be manipulated through the adjustments of the wavefronts, among which phason-like discontinuous dynamics is observed and quantitatively measured. Unlike optical quasicrystals shaped by electromagnetic fields, these spin-governed quasicrystals exhibit quasi-periodic properties of kinematic parameters, extending their potential applications to other physical systems. These findings hold promise for advancements in optical trapping, quasicrystal fabrication, and optical encryption systems.

## Teaser

The formation and manipulation of photonic spin quasicrystal is elucidated within a de Bruijn tiling like theoretical framework.

## INTRODUCTION

The discovery of quasicrystals, having long-range order without periodicity, has significantly influenced a wide range of fields (*1*–*8*). In the realm of photonics, the influence of distinct symmetries on wave propagation in quasi-periodic structures has given rise to numerous peculiar properties (*9*–*12*) and facilitated abundant optical functionalities (*13*–*19*). However, previous studies have primarily focused on the electric field patterns of quasi-periodic photonic systems, leaving quasi-periodic photonic spin textures largely unexplored. Recently, spin-orbit coupling in focused beams or evanescent waves has revealed a plethora of fascinating deep-subwavelength phenomena (*20*–*22*), and a variety of previously unidentified topological quasi-particles including skyrmions (*23*, *24*), merons (*25*), skyrmion lattices (*26*–*29*), meron lattices (*28*–*30*), hopfions (*31*), and topological spin defects (*32*) have been discovered in these photonic spin textures, which are also perceived as next-generation information carriers (*33*, *34*). The previous research of topological spin textures has been limited within the periodic conditions. In contrast, the study of arbitrary rotational symmetry holds a more generalized significance. Quasicrystals with higher symmetries provide more degrees of freedom and higher dimensionalities (*35*), which can produce more complex topologies (*36*, *37*) and reveal a range of unusual phenomena that are prohibited by the symmetry constraints present in periodic counterparts.

In this work, the formation mechanism of the quasi-periodic photonic spin textures was elucidated within a theoretical framework in compliance with the generalized multigrid method, which was presented by the mathematician N. G. de Bruijn and offered a reliable algorithm for generating the quasi-periodic tiling with various symmetries (*38*). This sheds light on a comprehensive understanding of quasi-periodic photonic systems with diverse symmetries, addressing a gap in previous studies that predominantly focused on photonic quasicrystals having specific symmetries. Furthermore, the configurations of the quasi-periodic photonic spin textures were manipulated by adjusting the wavefronts of the photonic systems, compatible with manipulating the quasi-periodic tiling configurations through adjustments of parallel line offsets in the generalized multigrid method. Within these configuration manipulations, phason-like discontinuous transports (*11*, *12*, *19*) were observed in quasi-periodic photonic systems, for which the displacements were quantitatively determined. The results of this work not only provide insights into the spin-orbit coupling with diverse symmetries under quasi-periodic conditions and the dynamics of quasi-periodic photonic spin textures but also have potential applications in the field of optical traps (*39*), quasicrystal fabrications through optical induction (*40*), and the optical encryption systems (*41*).

## RESULTS

### Formation mechanism of the photonic spin quasicrystal

**Fig. 1.**
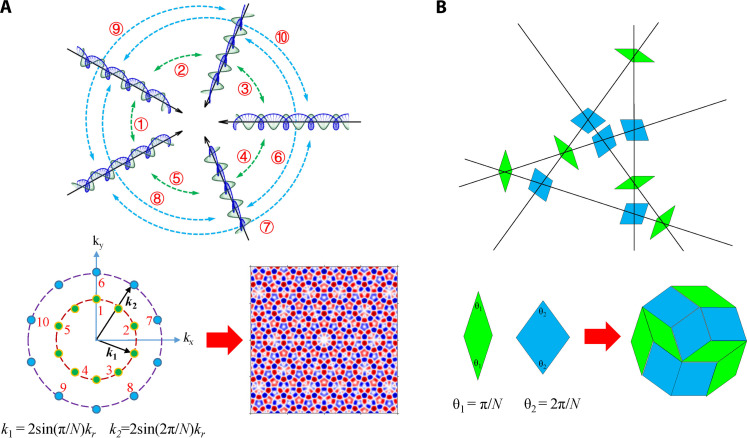
Formation mechanism of the photonic spin quasicrystal and its correspondence to the generation of the de Bruijn tiling. (**A**) Schematics of the interference of the evanescent waves (top), and the corresponding *S_z_* with *N* = 5 in the Fourier and the spatial domain (bottom). The correlations between each standing wave and the interaction of the evanescent waves are denoted by the numerical labels. (**B**) Typical region of the parallel lines (*N* = 5) with rhombuses constructed at each intersection (top), and the formation of de Bruijn tiling through assembling the rhombuses with different acute angles (bottom).

The generation of the quasi-periodic photonic spin textures was realized on the interference of the transverse magnetic evanescent waves. In this photonic system, the longitudinal component of the electric field is $E_{z}=\sum_{n=1}^{N}{\text{Ae}}^{{\mathrm{ik}}_{r}\left(x\text{cos}\frac{2n\pi }{N}+y\text{sin}\frac{2n\pi }{N}\right)}e^{-k_{z}z}$, with transverse components $E_{x}$ and $E_{y}$ satisfying $E_{x}=\frac{1}{k_{r}^{2}}\frac{\partial^{2}E_{z}}{\partial x\partial z}$ and $E_{y}=\frac{1}{k_{r}^{2}}\frac{\partial^{2}E_{z}}{\partial y\partial z}$ (*26*), where *A* is a constant, *N* is the number of evanescent waves, *k_r_* and *ik_z_* are the transverse and longitudinal wave-vector components. The spin angular momentum (SAM) is defined as ***S*** = Im[εE^*^ × ***E* +** μH^*^ × ***H***]/4ω (*42*), where ^*^ indicates complex conjugation; ω denotes the angular frequency of the electromagnetic field; ε and μ denote the permittivity and permeability of the medium, respectively. The longitudinal component of ***S*** can be mathematically represented as $S_{z}=\frac{\varepsilon }{2\omega }\sum s_{z}\prime \left(i,j\right)$ (see section S1), which involves a superposition of standing waves, with each standing wave being calculated as

$$s_{z}\prime =\text{sin}\left(\theta_{j}-\theta_{i}\right)\text{sin}\left[-k_{\alpha }\text{sin}\left(\frac{\theta_{i}+\theta_{j}}{2}\right)x+k_{\alpha }\text{cos}\left(\frac{\theta_{i}+\theta_{j}}{2}\right)y\right]$$ (1)

where θ*_i_* = 2*i*π/*N* and θ*_j_* = 2*j*π/*N* are the in-plane propagation angles of the *i*th and *j*th (*j > i*) evanescent wave, respectively; α = *j* − *i*; and *k*_α_ = 2sin(απ*/N*)*k_r_* is the wave vector of the standing wave. For example, the formation of the photonic spin quasicrystal with *N* = 5 is illustrated in Fig. 1A. At the top section of Fig. 1A, five evanescent waves propagating toward the center are illustrated, and the interactions between each pair of evanescent waves are represented by dashed arcs with numerical labels. As demonstrated in Eq. 1, each standing wave is formed by the interaction between the *i*th and *j*th evanescent waves, which also manifests as two points positioned opposite each other in the Fourier domain and labeled with the corresponding numbers, as shown in the bottom left corner of Fig. 1A. In the case of interference of evanescent waves with *N* = 5, there exist only two types of interactions: between the nearest and next-nearest evanescent waves, which are represented by α = 1 and α = 2, with green dash arc denoting the nearest interactions and blue dash arc indicating the next-nearest interactions. Consequently, there are two sets of wave vectors of the standing waves, *k*_1_ and *k*_2_. The formation mechanism of the photonic spin quasicrystal complies with the generation of quasi-periodic tiling based on the generalized multigrid approach (*38*). In the generalized multigrid method, the plane is divided into *N* sets of parallel lines that are evenly spaced, with each set oriented at an angle of 2*n*π/*N* (*n* = 1, 2, …, *N*). Degenerate points, where more than two lines intersect, are avoided by suitably choosing offsets of the lines in the direction normal to the lines. At each intersection between two lines, a rhombus is constructed with faces normal to those parallel lines. For example, a typical region of the parallel lines with *N* = 5 is illustrated in Fig. 1B. Note that there exist only two types of intersections, corresponding to thin and wide rhombus characterized by acute angles of π/5 and 2π/5, respectively. Once the rhombuses are assembled, a quasi-periodic tiling is generated, which manifests as a Penrose tiling (*43*) in the case of *N* = 5.

The correspondence between the generation of photonic spin quasicrystals and de Bruijn tiling is enumerated in Table 1, under the condition that *N* is an odd integer. Note that the interaction between evanescent waves resembles the intersection between parallel lines in the generalized multigrid method, where the number of types in both cases is equal to (*N* − 1)/2. Consequently, the wave vector of the standing waves can be compared to the rhombus in de Bruijn tiling, with the number of sets and the magnitude of the wave vector equal to the types of rhombuses and the sine of the acute angle of the rhombus, respectively, which are (*N* − 1)/2 and sin(απ*/N*).

**Table 1.** Correspondence between photonic spin quasicrystal and de Bruijn tiling.

Photonic spin quasicrystal	de Bruijn tiling	
Number of evanescent waves	Number of sets of parallel lines	*N*
Types of interaction between the evanescent waves	Types of intersection between the parallel lines	(*N* − 1)/2
Sets of wave vector of the spin textures	Types of rhombuses	(*N* − 1)/2
Magnitude of the wave vector of the spin textures (*k*_α_/2*k_r_*)	Sine of the acute angle of the rhombus	sin(απ/*N*)

The aforementioned model allows for the prediction of the number of sets and the magnitude of the wave vector, thereby enabling the calculation of the number and sizes of the sublattices comprising the topological photonic spin textures. This model not only accounts for photonic spin textures characterized by quasi-periodic structures but also encompasses periodic structures (see section S1), addressing ambiguities in previous research on skyrmion and meron lattices under periodic conditions (*28*, *44*). Among the calculated sublattices of the photonic spin texture, fractal structures can be observed (see section S2), exhibiting a self-similarity property also found in quasi-periodic tiling (*45*). Moreover, the model demonstrates distinct characteristics of quasi-periodic photonic spin texture in contrast to periodic circumstance. For example, previous research on periodic photonic spin texture revealed that the spin structure vanished when there was no external angular momentum (*l* = 0). However, the spin structure of quasi-periodic photonic spin texture persists even when *l* = 0, provided that *N* is an odd integer, which differs from the phenomenon observed in periodic photonic spin textures (see section S1).

### Configuration manipulation of photonic spin quasicrystals through wavefront adjustments

**Fig. 2.**
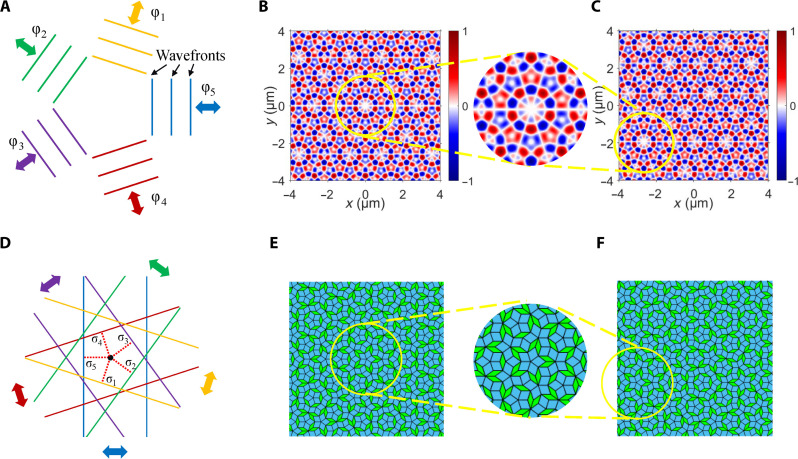
The de Bruijn tiling-style manipulation of the configurations of photonic spin quasicrystals through adjustments of the wavefronts. (**A**) Schematics of the adjustment of wavefronts of evanescent waves. (**B**) The calculated *S_z_* with *N* = 5 and the center marked by the yellow ring. (**C**) The calculated *S_z_* with additional phases (φ_1_, φ_2_, φ_3_, φ_4_, φ_5_) = (0, 0, 0.57π, 0, 0) applied on the evanescent waves. (**D**) Schematics of the adjustment of the offsets of parallel lines in the generalized multigrid method. (**E** and **F**) The de Bruijn tiling constructed from the parallel lines with offsets (E) (σ_1_, σ_2_, σ_3_, σ_4_, σ_5_) = (0.4, 0.4, 0.4, 0.4, 0.4) and (F) (σ_1_, σ_2_, σ_3_, σ_4_, σ_5_) = (0.4, 0.3, 0.2, 0.1, 0).

The configurations of the photonic spin quasicrystals can be manipulated by adjusting the wavefronts of the evanescent waves, as illustrated in Fig. 2A. The relative positions of the wavefronts can be altered by introducing additional phases to the evanescent waves, in which the phase applied on the *n*th evanescent wave is denoted as φ*_n_*. For example, the phases (φ_1_, φ_2_, φ_3_, φ_4_, φ_5_) = (0, 0, 0.57π, 0, 0) are applied to the quasi-periodic photonic spin texture with *N* = 5. The resulting photonic spin textures, both in the absence and presence of the applied phases, are illustrated in Fig. 2 (B and C). The center of the photonic spin texture without the applied phases is marked by the yellow ring. It is found that the phase manipulation results in a reconfiguration of the photonic spin quasicrystal, which is also equivalent to a displacement of the photonic spin texture. In this instance, the displacement occurs in the down-left direction. This approach parallels the field of mathematics, where configuration manipulations of the de Bruijn tiling can be achieved by adjusting the offsets of parallel lines, as illustrated in Fig. 2D. The coordinates of the parallel lines can be expressed as ${\vec{r}}_{n}\cdot {\vec{v}}_{n}=\left(Z-\sigma_{n}\right)d$, where ${\vec{r}}_{n}$ is the coordinate, ${\vec{v}}_{n}$ = [cos(2*n*π/*N*), sin(2*n*π/*N*)] is the normal vector, and σ*_n_* is the normalized offset of the *n*th set of parallel lines, respectively; *Z* is an integer; and *d* is the line spacing. When σ*_n_* is adjusted, the relative positions between different sets of parallel lines are changed, and the corresponding mathematical quasicrystal pattern is manipulated after each rhombus is constructed at each intersection of the grid lines and lastly assembled (*38*). For example, the de Bruijn tiling constructed from the parallel lines with offsets (σ_1_, σ_2_, σ_3_, σ_4_, σ_5_) = (0.4, 0.4, 0.4, 0.4, 0.4) is shown in Fig. 2E. If the offsets are adjusted to (σ_1_, σ_2_, σ_3_, σ_4_, σ_5_) = (0.4, 0.3, 0.2, 0.1, 0), the configuration of the de Bruijn tiling is manipulated, demonstrating a displacement in the down-left direction, as shown in Fig. 2F.

It was also found that, if a set of arbitrary phases are applied on the evanescent waves, then the quasi-periodic spin structure maintains its structural integrity except for a displacement, which is different from the behavior observed in periodic spin textures. This phenomenon can be explained through an analysis in the Fourier domain (see section S3). According to this analysis, the displacement of the quasi-periodic spin texture can be derived from the applied phase using the following equation (see section S4)

$$\begin{array}{c} \varphi_{n}=k_{n}\text{sin}\left(\frac{n}{N}\pi \right)\Delta x-k_{n}\text{cos}\left(\frac{n}{N}\pi \right)\Delta y+2m_{n}\pi +\varphi_{\text{re}}\\ \cdots \cdots \\ \varphi_{N}=\varphi_{\text{re}} \end{array}$$ (2)

where *n* = 1, 2, …, *N* − 1; *m_n_* is an integer; and φ_re_ is the reference phase. We first consider *N* = 5 as an example. The integer parameters *m*_1_, *m*_2_, *m*_3_, and *m*_4_ can be determined through numerical method, and the displacements can subsequently be calculated (see section S4). For example, when the phases (φ_1_, φ_2_, φ_3_, φ_4_, φ_5_) = (0.41π, 0.57π, 1.21π, 0.23π, 0) were applied to the evanescent waves, the displacement of the spin texture was calculated to be (∆*x*, ∆*y*) = (2.359 μm, −0.3512 μm). The *S_z_* distribution after applying the phases was calculated and is shown in fig. S6, demonstrating consistency with the theoretical prediction. For other odd values of *N*, the displacements of the spin textures can also be calculated from the applied phases by using Eq. 2 (see section S4). In this context, *N* = 3 is an exceptional case, for which the corresponding spin texture is periodic and the displacements can be determined analytically (see section S6).

If the additional phase is applied on only one of the evanescent waves, then it can be deduced from Eq. 2 that the spin texture exhibits one-dimensional displacement along the direction of the corresponding evanescent wave (see section S5). For example, when the phases (φ_1_, φ_2_, φ_3_, φ_4_, φ_5_) = (0, 0, 0, 0, φ_re_) were applied to the evanescent waves with *N* = 5 and φ_re_ = 0.5π, 0.57π, or 0.64π, the calculated one-dimensional displacements were ∆*x* = −1.1, 3.29, and −3.83 μm, respectively. The corresponding results of $S_{z}$ are shown in fig. S8. It was found that changing the phase of the evanescent wave results in a discontinuous translation of the spin texture, indicating the presence of phason-like dynamics within quasi-periodic systems. Phason excitations are unique to quasi-periodic systems, playing a role similar to phonons in periodic systems. According to the mathematical theory proposed by N. G. de Bruijn, quasi-periodic structures can be viewed as two-dimensional slices of hypercubic structures in higher dimensions. Consequently, the discontinuous translation of phasons in physical space corresponds to continuous dynamics occurring in higher-dimensional configuration space (*19*, *46*). The modulation of optical phason modes serves as a method for assessing the topological order of quasicrystals that display higher dimensional characteristics (*35*, *47*) and also holds promise for applications in the investigation of dislocation motion (*11*) and nonlinear interaction (*12*) within quasicrystals, as well as the manipulation of cold atoms through geometric pumping (*48*, *49*).

### Experimental observation of photonic spin quasicrystals

We used an in-house developed near-field imaging technique (*50*) to generate and map the spin structure of the quasi-periodic photonic spin textures. In this experimental scheme, the spin textures of surface plasmon polaritons (SPPs) sustained at the dielectric-metal interface of the sample were characterized using a dielectric nanoparticle as a near-field probe. A spatial light modulator (SLM) was used to generate *N* evenly distributed light spots by a 4*f* system, which were then focused via an oil immersion objective lens onto the sample (see Materials and Methods for the details of the experimental setup). The use of the SLM resulted in significantly smaller light spots on the back focal plane of the objective compared to those produced by intensity masks with apertures in previous studies (*28*, *51*). The incident energy was effectively used in this photonic system, and the effective scanning scope was expanded from 2 μm by 2 μm to 8 μm by 8 μm. Furthermore, the phase profile of the SLM can be manipulated by a computer to change the symmetry of the photonic spin textures, which is significantly more convenient than substituting intensity masks with varying apertures.

**Fig. 3.**
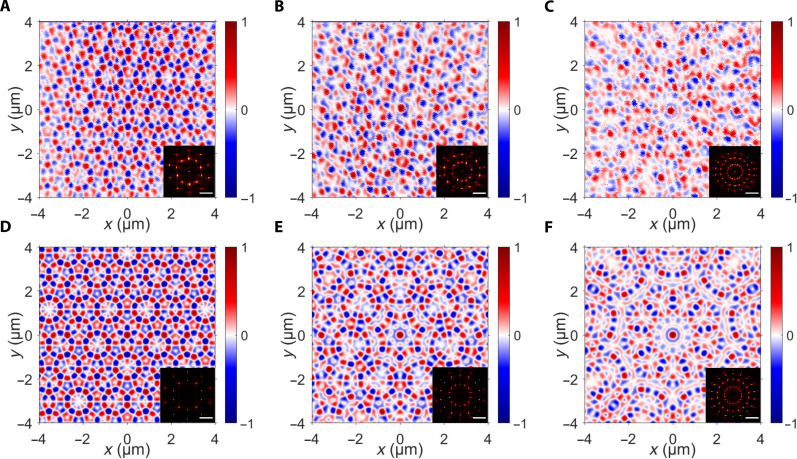
Measurement of photonic spin quasicrystals with diverse symmetries. (**A** to **C**) The measured longitudinal component of SAM with *l* = 1 and (A) *N* = 5, (B) *N* = 7, and (C) *N* = 9. The corresponding Fourier transform results are shown in the insets. (**D** to **F**) The corresponding calculated results of the spin textures. The scale bar in the insets is *k_r_* in the Fourier domain.

The measured longitudinal component of SAM for different values of *N* (*N* = 5, *N* = 7, and *N* = 9) and the phases of φ*_n_* = 2π*ln*/*N* with *l* = 1 is shown in Fig. 3 (A to C). The distributions of the measured quasi-periodic spin texture demonstrate *N*-fold rotational symmetry and correspond well to the calculated results for respective symmetries (Fig. 3, D to F). The Fourier transform results are shown in the insets of Fig. 3. Except the central point influenced by the background illumination, the experimental results demonstrate (*N* − 1)/2 sets and a total of *N*^2^ − *N* points for various *N* values and are consistent with the theoretical prediction (see section S1). It is observed that the discrepancy between the experimental and theoretical results increases with larger values of *N*, which may be attributed to two factors. First, the scanning scope for effectively illustrating the long-range order of the spin textures with larger *N* needs to be greater, and, therefore, within the same scanning scope, the discrepancy between the experimental results and theoretical calculations appears to be more pronounced for larger *N*. Second, as *N* increases, the number of the sublattices of spin textures also increases, potentially resulting in a greater degree of discrepancy between the experimental results and theoretical calculations.

**Fig. 4.**
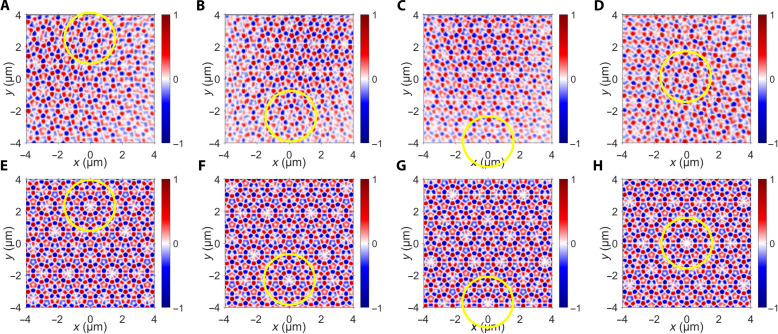
Phason-like discontinuous dynamics with wavefront modulations. (**A** to **D**) The measured longitudinal component of SAM with *N* = 5 and (A) *l* = 2, (B) *l* = 3, (C) *l* = 4, and (D) *l* = 5. (**E** to **H**) The corresponding calculated results of the spin textures.

Moreover, $S_{z}$ for *N* = 5 and spiral phases with different values of *l* (*l* = 2, *l* = 3, *l* = 4, and *l* = 5) were measured (Fig. 4, A to D) and consistent with the calculation results (Fig. 4, E to H). In this context, the spiral phase with *l* = 5 is equivalent to the situation where no extra phase is present. By manipulating the values of *l*, different phases were applied to adjust the wavefronts of the evanescent waves, while the photonic spin textures maintained structural integrity except for different displacements. These displacements can be obtained from Eq. 2 and were calculated to be ∆*x* = 0 μm and ∆*y* = 2.3196, −2.3196, −3.7371, and 0 μm, respectively, for *l* = 2, *l* = 3, *l* = 4, and *l* = 5.

## DISCUSSION

In summary, we provided a comprehensive explanation of the formation and manipulation mechanisms of photonic spin quasicrystals exhibiting diverse symmetries, all within a theoretical framework compatible to the principles of mathematical quasicrystals. It was found that adjusting the wavefronts of evanescent waves resulted in a discontinuous translation of the quasi-periodic spin texture, which was indicative of the phason-like dynamics and quantitatively determined within the proposed theoretical framework. The results of this work have potential applications in a wide range of fields. If the optical induction material is applied within optical quasicrystal pattern generated by interference of evanescent waves (*52*, *53*), then the quasicrystal fabrication can be facilitated by the method in this work, which has the advantage of enabling the fabrication of quasicrystal with diverse configurations through the manipulation of the applied phases. In addition, the theoretical framework in this work not only is limited in the evanescent wave but also can be extended to the electromagnetic waves in free space. The discontinuous displacement of the phason dynamics can be encoded and deciphered by the relevant quasicrystal algorithm, and, therefore, the method in this work can be applied in the optical encryption (*41*). Moreover, the SAM is closely related to the Poynting vector and the optical torque (*54*), and, therefore, the generation of quasi-periodic spin texture contributes to manipulation of nanoparticles in optical trapping with more degrees of freedom.

Furthermore, the superposition of the evanescent optical vortices on each lattice points of photonic metasurfaces has been shown to be equivalent to the interference of evanescent waves, if the incident wave vector can match the reciprocal vectors of the photonic metasurfaces (*28*, *29*). Consequently, in future research, the theoretical framework in this work can be applied in the investigation of the photonic spin textures of the quasicrystal metasurfaces with diverse symmetries, for which the optical modes are not only determined by the global symmetry of the photonic quasicrystal but also influenced by the local symmetry of the anisotropic meta-atoms on the quasicrystal lattice points, facilitating the functionalities including optical spin-Hall effect (*55*), manipulation of second harmonic generation radiation (*56*), and generation of holography (*57*).

It is worth noting that our proposed theoretical framework can be extended to other physical systems. It has been demonstrated that the SAM in electromagnetic guided waves can be derived from the kinetic momentum as $S=\frac{1}{2k^{2}}\nabla \times \Pi$, without the need for priori information of the electric and magnetic fields (*54*, *58*). This spin-momentum locking property has shown its universality in various types of fields, such as fluid (*59*), elastic (*60*), acoustic (*61*), and gravitational waves (*62*), which can be used to construct diverse topological spin structures, including Möbius-strip (*63*) and skyrmions (*64*–*66*) in a variety of classical waves. In the future, quasi-periodic spin textures are expected to be constructed based on our theoretical framework in various physical systems. This development is notable for the exploration of wave-matter interactions and the manipulation of the spin degrees of freedom in interdisciplinary research.

## MATERIALS AND METHODS

### Experimental setup

In the previous studies of the periodic photonic spin textures of SPPs (*28*, *51*), the incident beam was modulated with intensity masks composed of apertures to break the rotational symmetry and generate the interference of the evanescent waves, which blocked most of the incident energy with only a small portion passing through the apertures. Under such conditions, the angles of the apertures were designed to be larger than 10° to maintain a relative high signal-to-noise ratio of the mapping results, which determined the effective scanning scope of the spin textures to be smaller than 2 μm by 2 μm in the previous studies, and it is not enough to demonstrate the long-range order of the quasi-periodic photonic spin texture in this work. To expand the scanning scope, a SLM was used to generate *N* evenly distributed light spots for interference of the evanescent waves. The experimental setup is shown in fig. S10. After passing through a telescope system, an incident laser beam with wavelength of 632.8 nm illuminated the reflecting liquid SLM, which provided the phase profile of the interference of *N* waves. The Fourier transform of *e*^*i*Φ^ manifests as *N* spots that are evenly distributed on a ring. In the experiment, the modulated incident field was weakly focused by a lens with focal length of 500 mm to perform the Fourier transform. The *N* evenly distributed light spots were generated at the focal point and then were focused by a 4*f* system onto the back focal plane of the oil immersion objective [Olympus, numerical aperture (NA) = 1.49, 100×]. A combination of a linear polarizer (LP) and an *m* = 1 vortex wave plate was used to turn the incident field into radial polarized. The incident beam was tightly focused by the oil immersion objective to excite the SPPs at the air/gold interface of the sample with 50-nm-thick gold film deposited on a glass substrate. By adjusting the wave vectors in the phase profile, the *N* evenly distributed light spots matched the dark ring that indicated the excitation of the SPPs. In this instance, *N* evanescent waves were generated at the air/gold interface.

A polystyrene nanoparticle with a radius of 160 nm was immobilized on the gold film by the 4-mercaptobenzoic acid molecular linker to scatter the transverse component of the near-field SPPs to the far-field for collection. The scattering radiation was collected by an objective (Olympus, NA = 0.7, 60×), and the right and left circular polarized components of the scattering radiation were filtered out by a quarter-wave plate and a LP. After polarization selection, the intensity of light of each circular polarized component is measured by the photomultiplier tube (Hamamatsu R12829). The nanoparticle-on-film sample was fixed on a Piezo scanning stage (Physik Instrumente, P-545) to perform the two-dimensional scanning, and the spin structure can be mapped.
