# Supplementary material for: Photonic quasicrystal of spin angular momentum
Source: Sci Adv. 2025 Apr 30;11(18):eadv3938. doi: 10.1126/sciadv.adv3938 (PMC12042881; doi:10.1126/sciadv.adv3938)
Supplement: Supplementary file 1 — Supplementary Text Figs. S1 to S11 [file sciadv.adv3938_sm.pdf]

Science Advances
AAAS

Supplementary Materials for

Photonic quasicrystal of spin angular momentum

Min Lin *et al.*

Corresponding author: Luping Du, lpdu@szu.edu.cn

Sci. Adv. **11**, eadv3938 (2025)
DOI: 10.1126/sciadv.adv3938

This PDF file includes:

Supplementary Text
Figs. S1 to S11

Session 1: Derivation of the photonic spin textures

The electric field of the interference of the evanescent waves is expressed as

$$\mathbf{E}=\begin{pmatrix}E_x\\E_y\\E_z\end{pmatrix}=\begin{pmatrix}-i\frac{k_z}{k_r}\sum_{n=1}^{N}A\cos\theta_n e^{ik_r(x\cos\theta_n+y\sin\theta_n)}\\-i\frac{k_z}{k_r}\sum_{n=1}^{N}A\sin\theta_n e^{ik_r(x\cos\theta_n+y\sin\theta_n)}\\\sum_{n=1}^{N}Ae^{ik_r(x\cos\theta_n+y\sin\theta_n)}\end{pmatrix}e^{-k_z z}, \tag{S1}$$

where A is a constant, N is the number of evanescent waves, k_r and ik_z are the transverse and longitudinal wave-vector components, $\theta_n=2n\pi/N$ is the in-plane propagation angle of the nth evanescent wave. The SAM is defined as $\boldsymbol{S}=\mathrm{Im}[\varepsilon\boldsymbol{E}^*\times\boldsymbol{E}+\mu\boldsymbol{H}^*\times\boldsymbol{H}]/4\omega$, and therefore the longitudinal component of $\boldsymbol{S}$ is calculated as $S_z=\frac{\varepsilon}{2\omega}(\frac{k_r}{k_z})^2\mathrm{Im}(E_x^*E_y)$. S_z can be mathematically represented as $S_z=\frac{\varepsilon}{2\omega}\sum s_z(i,j)$, and we obtain

$$\begin{aligned}s_z&=\mathrm{Im}\left[\cos\theta_i e^{-ik_r(x\cos\theta_i+y\sin\theta_i)}\cdot\sin\theta_j e^{ik_r(x\cos\theta_j+y\sin\theta_j)}\right]\\&=\cos\theta_i\sin\theta_j\sin\left[-k_\alpha\sin\left(\frac{\theta_i+\theta_j}{2}\right)x+k_\alpha\cos\left(\frac{\theta_i+\theta_j}{2}\right)y\right]\end{aligned}, \tag{S2}$$

where $\alpha=j-i$ ($j>i$) and $k_\alpha=2\sin(\alpha\pi/N)k_r$. Equation (S2) is the result of the interaction between E_x of the ith evanescent wave and E_y of the jth evanescent wave. Note that when $i=j$, $s_z=0$. On the other hand, when $i\neq j$, $s_z(i,j)$ and $s_z(j,i)$ have the same wave-vector and can be degenerated into one term as $s_z'(i,j)=s_z(i,j)+s_z(j,i)$, which is calculated as

$$s_z'=\sin(\theta_j-\theta_i)\sin\left[-k_\alpha\sin\left(\frac{\theta_i+\theta_j}{2}\right)x+k_\alpha\cos\left(\frac{\theta_i+\theta_j}{2}\right)y\right]. \tag{S3}$$

Considering the case of $i=j$ and $i\neq j$, the number of standing waves, represented as s_z', can be determined as $(N^2-N)/2$, when N is an odd integer.

If N is an even integer, the case of counter propagation of evanescent waves must be considered. In this instance, $s_z'=0$ when $\theta_j-\theta_i=\pi$. Moreover, $s_z'(i+N/2, j+N/2)$ has the same wave-vector as $s_z'(i,j)$, and they can be further degenerated into one term as $s_z''(i,j)$, which is calculated as 0, and therefore the spin texture is absent for even N.

If a spiral phase of $\varphi_n=2\pi ln/N$ is applied on the system, $s_z''(i,j)$ for even N is calculated as

$$s_z''=2\sin(\theta_j-\theta_i)\sin\left[l(\theta_j-\theta_i)\right]\cos\left[-k_\alpha\sin\left(\frac{\theta_i+\theta_j}{2}\right)x+k_\alpha\cos\left(\frac{\theta_i+\theta_j}{2}\right)y\right]. \tag{S4}$$

In this instance, the number of standing waves is $(N^2-2N)/4$ for even N. Considering the types of the interaction between the evanescent waves, the number of values of k_α is $(N-1)/2$ and $(N-2)/2$ for odd N and even N with spiral phase respectively.

According to equation (S4), it is found that if N is an even integer, $S_z=0$ when $l=0$. It indicates that, in the absence of external angular momentum, the spin textures vanish for even N. However, if N is an odd integer, $S_z\neq0$ when $l=0$, which is different from the phenomenon for even N. For example, S_z for $N=5(l=0)$ and $N=6(l=1)$ was calculated along with its Fourier transform, as depicted in Fig. S1. For one standing wave, it corresponds to two points positioned opposite each other in the Fourier domain. As shown in Fig. S1 (c, d), the number of points in the Fourier domain are 20 and 12 for $N=5$ and $N=6$ respectively, and the sets of points classified by wave-vectors are both 2 for $N=5$ and $N=6$, which are consistent with the theoretical prediction. For $N=6$, the photonic skyrmion lattice consists of two sublattices with different wave-vectors, which was observed but the formation mechanism was ambiguous in the previous studies (*28*,*44*). In this work, the number of sublattices can be predicted by the model proposed above.

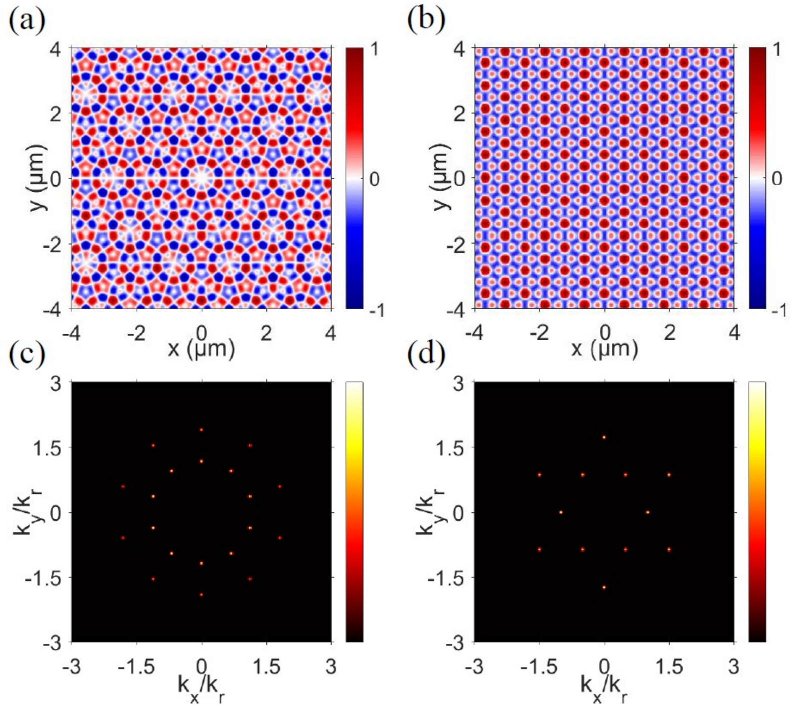

Fig. S1: Comparison between the photonic spin textures and their Fourier transform with odd and even value of N. (a, b) The longitudinal component of SAM with (a) $N=5$ and $l=0$, and (b) $N=6$ and $l=1$. (c, d) The corresponding Fourier transform results of S_z.

The correspondence between the generation of photonic spin textures and de Bruijn tiling with even N is examined in the following discussion. For example, the formation of the photonic spin texture with N=6 is illustrated in Fig. S2 (a). There are two types of interactions: between the nearest and next-nearest evanescent waves, which are denoted by green and blue dash arcs respectively. In this instance, the interaction between the next-next-nearest evanescent waves is absent, as the angle between two evanescent waves is 180°, resulting in a calculated value of the spin texture of 0 from equation (S4). The corresponding de Bruijn tiling with N=6 is shown in Fig. S2 (b), in which there are 3 sets of parallel lines with each set oriented at an angle of $2n\pi/N$. In this instance, only a type of rhombus is constructed at each intersection between two lines, and a hexagonal crystal is formed. In the context of mathematical tiling, an angle of 180° is also absent in rhombuses, which is similar to the formation of photonic spin textures. However, in the generation of the photonic spin texture, the interactions between the nearest and next-nearest evanescent waves correspond to the angle of 60° and 120°, representing two distinct types of interactions. While in the context of the mathematical tiling, these two angles correspond to only one type of rhombuses. Therefore, the number of sets of wave-vectors of the photonic spin textures is different from the number of types of rhombuses of the mathematical tiling when N is an even integer. The preceding analysis pertains to periodic structures, and the subsequent examination of quasicrystals with even values of N adheres to the same principles. For example, the formation of the photonic spin texture with N=12 is demonstrated in in Fig. S2 (c) and it is found that there are 5 types of interactions. This figure presents only a selection of representative interactions for the purpose of simplification, omitting the depiction of all possible interactions. The corresponding de Bruijn tiling with N=12 is illustrated in Fig. S2 (d), in which there are 3 types of rhombuses, and a hexagonal quasicrystal is formed when the rhombuses at each intersection are assembled. For the photonic spin textures with even N, the number of sets of wave-vectors is $(N-2)/2$, which is determined by the number of types of interaction between the evanescent waves. While the number of types of rhombuses for the mathematical quasicrystals with even N is given by the expression $\lfloor N/4 \rfloor$, which is determined by the number of acute angles that can be formed through the intersection of the grid lines. Although the number of sets of wave-vectors of the photonic spin textures is different from the number of types of rhombuses of the mathematical tiling when N is an even integer, the photonic spin textures, including crystals and quasicrystals with even N can still be described by the theoretical framework in this work.

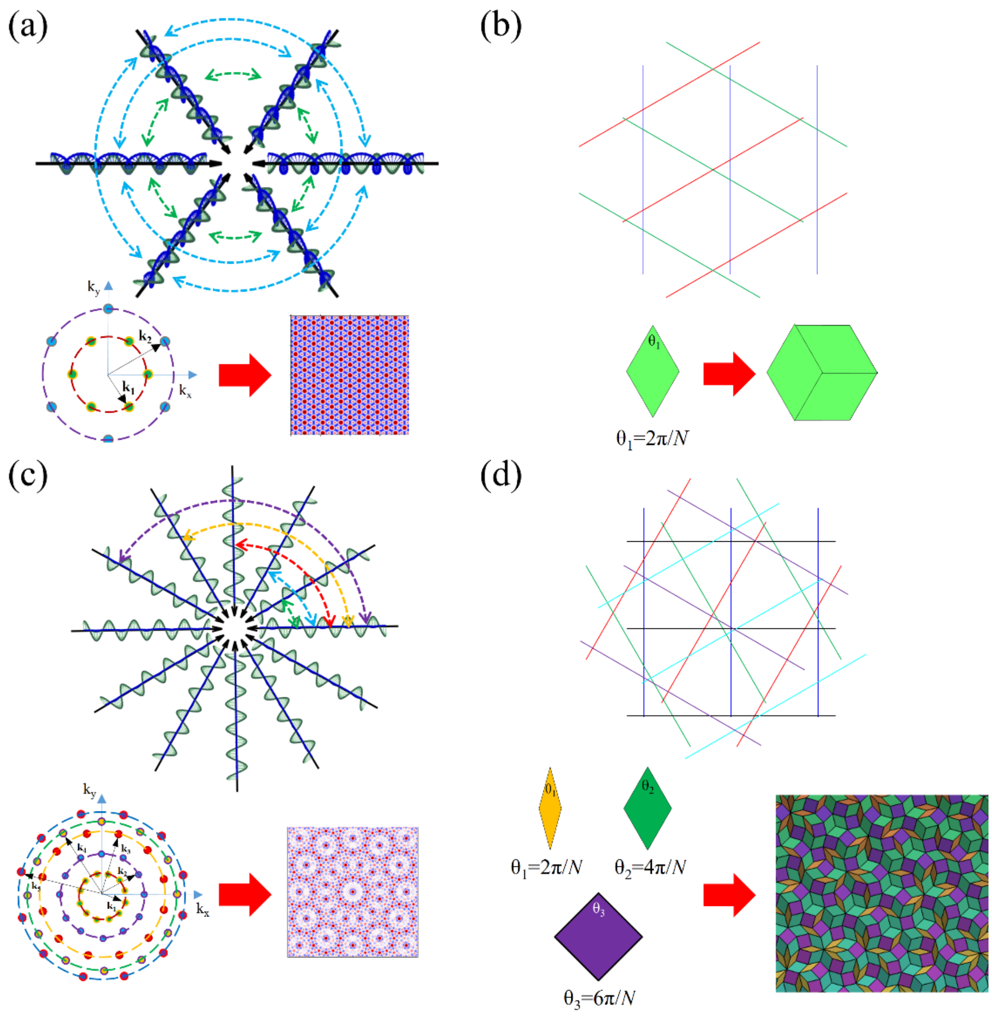

Fig. S2: Formation mechanism of the photonic spin texture and its correspondence to the generation of the de Bruijn tiling with even value of *N*. (a, c) Schematics of the interference of the evanescent waves (top), and the corresponding S_z in the Fourier and the spatial domain (bottom) with (a) N=6 and l=1, and (c) N=12 and l=1. (b, d) Typical region of the parallel lines with rhombuses constructed at each intersection (top), and the formation of de Bruijn tiling through assembling the rhombuses with different acute angles (bottom) with (b) N=6 and (d) N=12.

Session 2: Fractal structure of the photonic spin texture

The aforementioned model reveals that the photonic spin texture exhibits a fractal configuration. For example, two sublattices with different wave-vectors of the photonic spin texture for N=5 and l=0 were calculated and shown in Fig. S3 (a) and (b). The sublattice with the wave-vector of k_2 exhibits an identical spin configuration to that of the sublattice with k_1, albeit

with a smaller size and opposite sign. When N is an odd integer, this phenomenon can be accounted for through substituting different values of α into equation (S3).

If N is an even integer, a fractal structure is formed with the spin structure slightly different from the case for odd N. For example, two sublattices with different wave-vectors of the photonic spin texture for N=6 and l=1 were calculated and shown in Fig. S3 (c, d). The sublattice with the wave-vector of k_2 can be regarded as a result of π/6 rotation for the sublattice with k_1 together with $1/\sqrt{3}$ scale shrink. This phenomenon can be accounted for through substituting different values of α into equation (S4).

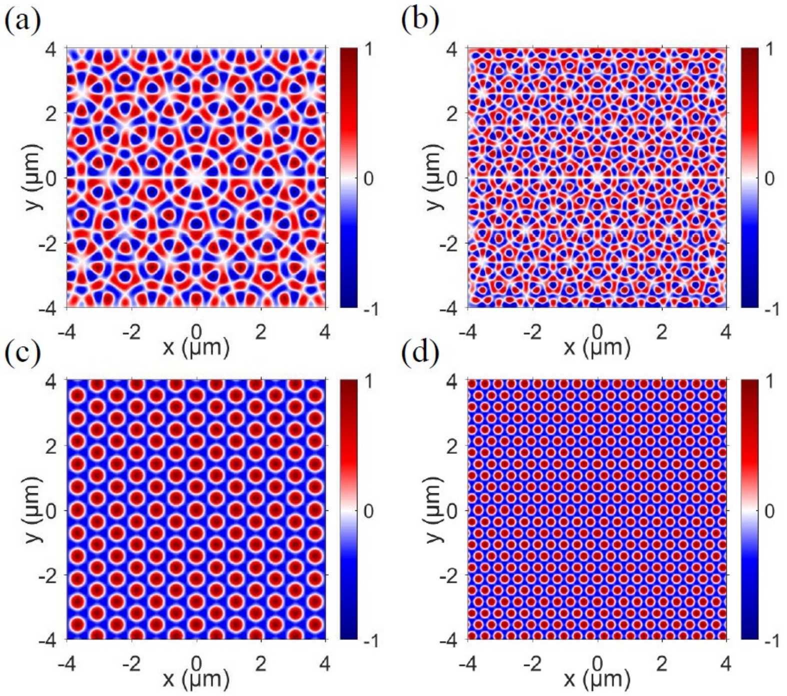

Fig. S3: Fractal structure of the photonic spin textures. (a, b) The summation of s_z' with N=5 and l=0 and (a) the wave-vector of $k_1=2\sin(\pi/5)k_r$, and (b) $k_2=2\sin(2\pi/5)k_r$. (c, d) The summation of s_z'' with N=6 and l=1 and (c) the wave-vector of $k_1=2\sin(\pi/6)k_r$, and (d) $k_2=2\sin(2\pi/6)k_r$.

Session 3: Robustness of the quasi-periodic photonic spin texture against the applied phase

For odd N, the spin texture is robust against the additional phase applied on the evanescent waves, which is different from the phenomenon for even N. For example, the longitudinal component of SAM for N=5(l=0) and N=6(l=1) with arbitrary phases (φ_1, φ_2, φ_3, φ_4, φ_5)=(0.30π, 0.11π, 0.56π, 0.28π, 0.42π) and (φ_1, φ_2, φ_3, φ_4, φ_5, φ_6)=(0.11π, 0.25π, 0.81π, 0.45π, 0.59π, 0.32π) were calculated and shown in Fig. S4 (a, b). It is found that the spin texture for N=5 maintains

structural integrity except for displacement, while the spin texture for N=6 is disturbed by the applied phases. This phenomenon can be accounted for through an analysis in the Fourier domain. For odd N, the Fourier transform of the spin texture can be expressed as summation of $F[s_z']$, which is calculated as

$$\begin{aligned} F[s_z'] &= i\pi\sin(\theta_j-\theta_i)\delta\left(k_x-k_\alpha\sin\left(\frac{\theta_i+\theta_j}{2}\right),k_y+k_\alpha\cos\left(\frac{\theta_i+\theta_j}{2}\right)\right) \\ &-i\pi\sin(\theta_j-\theta_i)\delta\left(k_x+k_\alpha\sin\left(\frac{\theta_i+\theta_j}{2}\right),k_y-k_\alpha\cos\left(\frac{\theta_i+\theta_j}{2}\right)\right) \end{aligned}, \quad \text{(S5)}$$

where the two delta functions correspond to the two points in the Fourier domain. If the phases applied on the ith and j^{th} evanescent waves are denoted as φ_i and φ_j, $F[s_z']$ with applied phase is expressed as

$$\begin{aligned} F[s_z'] &= i\pi\sin(\theta_j-\theta_i)e^{i(\varphi_i-\varphi_j)}\delta\left(k_x-k_\alpha\sin\left(\frac{\theta_i+\theta_j}{2}\right),k_y+k_\alpha\cos\left(\frac{\theta_i+\theta_j}{2}\right)\right) \\ &-i\pi\sin(\theta_j-\theta_i)e^{i(\varphi_j-\varphi_i)}\delta\left(k_x+k_\alpha\sin\left(\frac{\theta_i+\theta_j}{2}\right),k_y-k_\alpha\cos\left(\frac{\theta_i+\theta_j}{2}\right)\right) \end{aligned}. \quad \text{(S6)}$$

It is found that the introduction of the additional phase will not change the amplitude of the two delta functions for odd N, and therefore the Fourier transform result in Fig. S4 (c) is the same as Fig. S1 (c). In this instance, the spin texture maintains its structural integrity. For even N, the Fourier transform of the spin texture can be expressed as summation of $F[s_z'']$, which is calculated as

$$\begin{aligned} F[s_z''] &= 2\pi\sin(\theta_j-\theta_i)\sin\left[l(\theta_j-\theta_i)\right]\delta\left(k_x-k_\alpha\sin\left(\frac{\theta_i+\theta_j}{2}\right),k_y+k_\alpha\cos\left(\frac{\theta_i+\theta_j}{2}\right)\right) \\ &+2\pi\sin(\theta_j-\theta_i)\sin\left[l(\theta_j-\theta_i)\right]\delta\left(k_x+k_\alpha\sin\left(\frac{\theta_i+\theta_j}{2}\right),k_y-k_\alpha\cos\left(\frac{\theta_i+\theta_j}{2}\right)\right) \end{aligned}. \quad \text{(S7)}$$

After the additional phases are introduced, $F[s_z'']$ is expressed as

$$\begin{aligned} F[s_z''] &= i\pi\sin(\theta_j-\theta_i)\left(e^{i\left[l(\theta_i-\theta_j)+\varphi_i-\varphi_j\right]}-e^{i\left[l(\theta_j-\theta_i)+\varphi_{j+N/2}-\varphi_{i+N/2}\right]}\right)\delta\left(k_x-k_\alpha\sin\left(\frac{\theta_i+\theta_j}{2}\right),k_y+k_\alpha\cos\left(\frac{\theta_i+\theta_j}{2}\right)\right) \\ &-i\pi\sin(\theta_j-\theta_i)\left(e^{i\left[l(\theta_j-\theta_i)+\varphi_j-\varphi_i\right]}-e^{i\left[l(\theta_i-\theta_j)+\varphi_{i+N/2}-\varphi_{j+N/2}\right]}\right)\delta\left(k_x+k_\alpha\sin\left(\frac{\theta_i+\theta_j}{2}\right),k_y-k_\alpha\cos\left(\frac{\theta_i+\theta_j}{2}\right)\right) \end{aligned}. \quad \text{(S8)}$$

It is found that the applied phase changes the amplitude of the delta function from $2\pi\sin(\theta_j-\theta_i)\sin\left[l(\theta_j-\theta_i)\right]$ to $\pi\sin(\theta_j-\theta_i)\sqrt{2-2\cos\left[2l(\theta_j-\theta_i)+\varphi_j-\varphi_i+\varphi_{j+N/2}-\varphi_{i+N/2}\right]}$. In this

instance, some points of the Fourier transform result may disappear if the amplitude of the delta function is zero, as shown in Fig. S4 (d), and the corresponding spin texture will be disturbed.

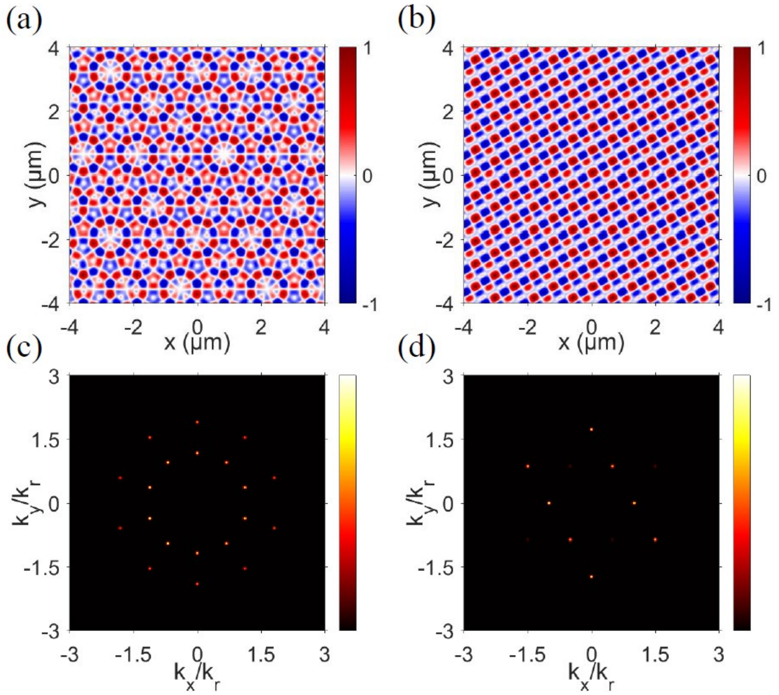

Fig. S4: Robustness of the photonic spin textures against the applied phase. (a, b) The longitudinal component of SAM with arbitrary phase and (a) N=5 and l=0, (b) N=6 and l=1. (c, d) The corresponding Fourier transform results of S_z.

Session 4: Derivation of the relation between the applied phase and the displacement

For odd N, if the spin texture has a 2D displacement of Δx and Δy, the Fourier transform of s_z' is obtained as

$$\begin{aligned} &F\left[s_z'\right](x-\Delta x, y-\Delta y) \\ &= i\pi \sin\left(\theta_j-\theta_i\right) e^{i\left(-k_\alpha \sin\left(\frac{\theta_i+\theta_j}{2}\right)\Delta x + k_\alpha \cos\left(\frac{\theta_i+\theta_j}{2}\right)\Delta y\right)} \delta\left(k_x - k_\alpha \sin\left(\frac{\theta_i+\theta_j}{2}\right), k_y + k_\alpha \cos\left(\frac{\theta_i+\theta_j}{2}\right)\right). \\ &-i\pi \sin\left(\theta_j-\theta_i\right) e^{i\left(k_\alpha \sin\left(\frac{\theta_i+\theta_j}{2}\right)\Delta x - k_\alpha \cos\left(\frac{\theta_i+\theta_j}{2}\right)\Delta y\right)} \delta\left(k_x + k_\alpha \sin\left(\frac{\theta_i+\theta_j}{2}\right), k_y - k_\alpha \cos\left(\frac{\theta_i+\theta_j}{2}\right)\right) \end{aligned} \quad \text{(S9)}$$

It is found that equation (S6) is equivalent to equation (S9) if $\varphi_i = k_i \sin\left(\frac{i\pi}{N}\right)\Delta x - k_i \cos\left(\frac{i\pi}{N}\right)\Delta y$ and $\varphi_j = k_j \sin\left(\frac{j\pi}{N}\right)\Delta x - k_j \cos\left(\frac{j\pi}{N}\right)\Delta y$ are satisfied, which is verified below

$$
\begin{aligned}
\varphi_i - \varphi_j &= \left[k_i \sin\left(\frac{i\pi}{N}\right) - k_j \sin\left(\frac{j\pi}{N}\right)\right]\Delta x + \left[k_j \cos\left(\frac{j\pi}{N}\right) - k_i \cos\left(\frac{i\pi}{N}\right)\right]\Delta y \\
&= \left[\cos\left(\frac{2j\pi}{N}\right) - \cos\left(\frac{2i\pi}{N}\right)\right]k_r \Delta x + \left[\sin\left(\frac{2j\pi}{N}\right) - \sin\left(\frac{2i\pi}{N}\right)\right]k_r \Delta y \\
&= 2\sin\left(\frac{i+j}{N}\pi\right)\sin\left(\frac{i-j}{N}\pi\right)k_r \Delta x - 2\cos\left(\frac{i+j}{N}\pi\right)\sin\left(\frac{i-j}{N}\pi\right)k_r \Delta y \\
&= -k_\alpha \sin\left(\frac{\theta_i + \theta_j}{2}\right)\Delta x + k_\alpha \cos\left(\frac{\theta_i + \theta_j}{2}\right)\Delta y
\end{aligned}
\quad . \qquad \text{(S10)}
$$

In this instance, the 2D displacements of the spin textures can be obtained from the additional phase applied on the system through the equation

$$
\begin{aligned}
&\varphi_n = k_n \sin\left(\frac{n}{N}\pi\right)\Delta x - k_n \cos\left(\frac{n}{N}\pi\right)\Delta y + 2m_n\pi + \varphi_{re} \\
&\cdots\cdots \\
&\varphi_N = \varphi_{re}
\end{aligned}
\quad , \qquad \text{(S11)}
$$

where n=1, 2, … N−1, m_n is an integer, and φ_{re} is the reference phase. We first consider N=5 as an example, and equation (S11) can be expressed as

$$
\begin{aligned}
&\varphi_1 = k_1 \sin\frac{\pi}{5}\Delta x - k_1 \cos\frac{\pi}{5}\Delta y + 2m_1\pi + \varphi_{re} \\
&\varphi_2 = k_2 \sin\frac{2\pi}{5}\Delta x - k_2 \cos\frac{2\pi}{5}\Delta y + 2m_2\pi + \varphi_{re} \\
&\varphi_3 = k_2 \sin\frac{2\pi}{5}\Delta x + k_2 \cos\frac{2\pi}{5}\Delta y + 2m_3\pi + \varphi_{re} \\
&\varphi_4 = k_1 \sin\frac{\pi}{5}\Delta x + k_1 \cos\frac{\pi}{5}\Delta y + 2m_4\pi + \varphi_{re} \\
&\varphi_5 = \varphi_{re}
\end{aligned}
\quad . \qquad \text{(S12)}
$$

From equation (S12), we obtain

$$
\begin{aligned}
\varphi_2 - \varphi_3 + 2(m_3 - m_2)\pi &= -k_1 \Delta y \\
\varphi_1 - \varphi_4 + 2(m_4 - m_1)\pi &= -k_2 \Delta y
\end{aligned}
\quad , \qquad \text{(S13)}
$$

and

$$
\begin{aligned}
\varphi_2 + \varphi_3 - 2\varphi_{re} - 2(m_3 + m_2)\pi &= 2k_2 \sin\frac{2\pi}{5}\Delta x \\
\varphi_1 + \varphi_4 - 2\varphi_{re} - 2(m_4 + m_1)\pi &= 2k_1 \sin\frac{\pi}{5}\Delta x
\end{aligned}
\quad . \qquad \text{(S14)}
$$

From equations (S13) and (S14), we further obtain

$$
2\cos\left(\frac{2\pi}{5}\right)(m_4 - m_1) = (m_3 - m_2) + \frac{\varphi_2 - \varphi_3 + 2\cos\left(\frac{2\pi}{5}\right)(\varphi_4 - \varphi_1)}{2\pi} , \qquad \text{(S15)}
$$

and

$$4\cos^2\left(\frac{\pi}{5}\right)(m_4+m_1)=(m_3+m_2)+\frac{4\cos^2\left(\frac{\pi}{5}\right)(\varphi_1+\varphi_4-2\varphi_{re})-(\varphi_2+\varphi_3-2\varphi_{re})}{2\pi}. \quad \text{(S16)}$$

Let $p_1=m_4-m_1$, $q_1=m_3-m_2$, $p_2=m_4+m_1$, $q_2=m_3+m_2$. For equation (S15), we can find the point with integer coordinate of (p_1, q_1) which is closest to the line with slope of $2\cos\left(\frac{2\pi}{5}\right)$ and intercept of $-\frac{\varphi_2-\varphi_3+2\cos\left(\frac{2\pi}{5}\right)(\varphi_4-\varphi_1)}{2\pi}$ in a numerical way. For example, if the applied phases are (φ_1, φ_2, φ_3, φ_4, φ_5)=(0.41π, 0.57π, 1.21π, 0.23π, 0) and the allowed error of q_1 is set within 0.04, we can obtain the coordinate of (p_1, q_1) as (1,1), as shown in Fig. S5 (a). From equation (S16), we can obtain (p_2, q_2) as (−5, −13) in the same way, as shown in Fig. S5 (b).

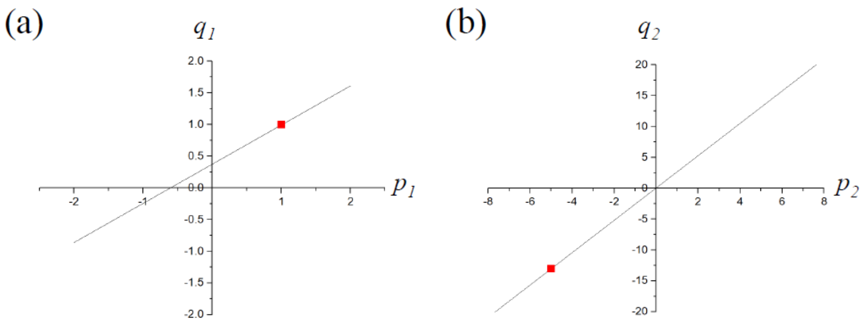

Fig. S5: Calculation of the integer parameters in equation (S12). (a) The line with slope of $2\cos(2\pi/5)$ and intercept of $\left[\varphi_3-\varphi_2+2\cos(2\pi/5)(\varphi_1-\varphi_4)\right]/2\pi$ and the point with the integer coordinate of (1,1). (b) The line with slope of $4\cos^2(\pi/5)$ and intercept of $\left[\varphi_2+\varphi_3-2\varphi_{re}-4\cos^2(\pi/5)(\varphi_1+\varphi_4-2\varphi_{re})\right]/2\pi$ and the point with the integer coordinate of (−5, −13).

After (p_1, q_1) and (p_2, q_2) are obtained, it is necessary to ensure that both p_1+p_2 and q_1+q_2 are even to obtain the integer values of m_n, which are m_1=−3, m_2=−7, m_3=−6, m_4=−2. Finally, the displacements are calculated as

$$\begin{aligned}\Delta x&=-\frac{2(m_4+m_1)\pi+2\varphi_{re}-\varphi_1-\varphi_4}{2k_1\sin\frac{\pi}{5}},\\ \Delta y&=-\frac{2(m_4-m_1)\pi+\varphi_1-\varphi_4}{k_2}\end{aligned} \quad \text{(S17)}$$

and we obtain Δx=2.359μm, Δy=−0.3512μm. As shown in Fig. S6 (a) and (b), S_z of the spin textures for N=5 applied with arbitrary phases (φ_1, φ_2, φ_3, φ_4, φ_5)=(0.41π, 0.57π, 1.21π, 0.23π, 0) is the same as the one with displacements of Δx=2.359μm and Δy=−0.3512μm.

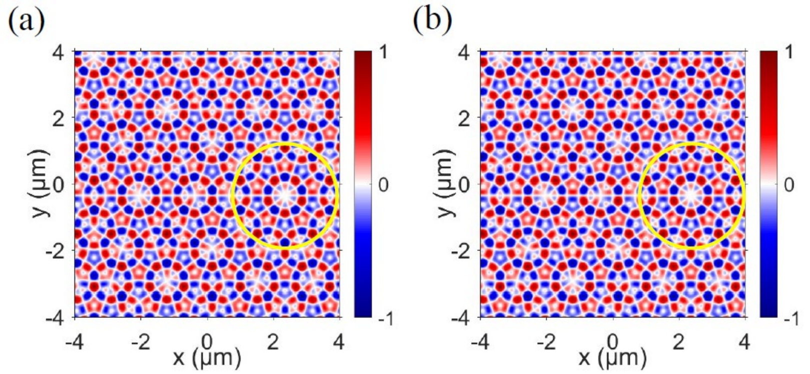

Fig. S6: Displacement of the photonic spin quasicrystals with *N*=5 under the applied phase. (a) The longitudinal component of SAM with N=5 and arbitrary phases (φ_1, φ_2, φ_3, φ_4, φ_5)=(0.41π, 0.57π, 1.21π, 0.23π, 0). (b) The longitudinal component of SAM with N=5 and displacements of Δx=2.359μm, Δy=−0.3512μm. The yellow rings mark the center of the photonic spin texture with N=5 and no applied phases.

For other values of odd N, the displacements of the spin textures can also be obtained from the applied phase by using equation (S11). For example, when N=7, if the applied arbitrary phases are (φ_1, φ_2, φ_3, φ_4, φ_5, φ_6, φ_7)=(0.41π, 0.57π, 1.21π, 0.23π, 0.55π, 0.37π, 0), we can obtain the integer parameters m_1=131, m_2=352, m_3=497, m_4=456, m_5=261, m_6=58 from equation (S11), and the displacements are calculated as Δx=−153.49μm, Δy=28.6μm. As shown in Fig. S7, it is found that S_z with applied arbitrary phases is the same as the one with displacement for N=7.

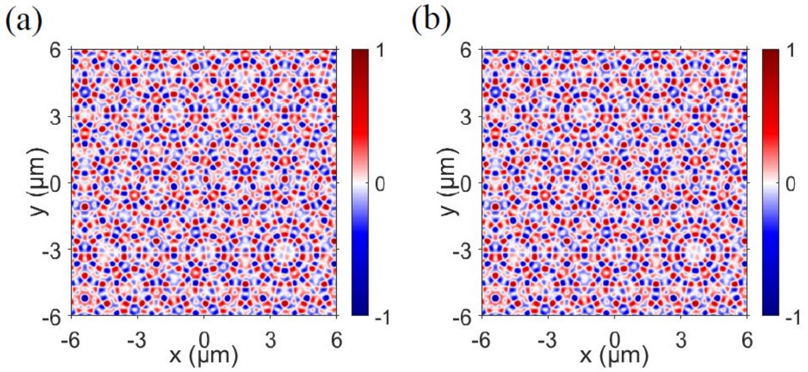

Fig. S7: Displacement of the photonic spin quasicrystals with *N*=7 under the applied phase. (a) The longitudinal component of SAM with *N*=7 and applied arbitrary phases (φ_1, φ_2, φ_3, φ_4, φ_5, φ_6, φ_7)=(0.41π, 0.57π, 1.21π, 0.23π, 0.55π, 0.37π, 0). (b) The longitudinal component of SAM with *N*=7 and displacements of Δx=−153.49μm, Δy=28.6μm.

Session 5: 1D displacement of the spin texture for additional phase applied on one evanescent wave

According to equation (S11), it is found that if the phase is only applied on the Nth evanescent waves, Δy=0 and the spin texture has 1D displacement along the x-axis. The in-plane propagation angle of the Nth evanescent wave is 0 and it is in the same direction as the 1D displacement of the spin texture. Therefore, due to the rotational symmetry, the spin texture has 1D displacement along the direction of the propagation of the evanescent wave with applied phase. For example, the phases (φ_1, φ_2, φ_3, φ_4, φ_5)=(0, 0, 0, 0, φ_{re}) are applied on the evanescent waves with *N*=5. In this instance, we obtain Δy=0, m_1=m_4, and m_2=m_3 from equation (S12). From equation (S14), we obtain

$$\begin{aligned}\varphi_{re}+2m_1\pi&=-k_1\sin\frac{\pi}{5}\Delta x\\ \varphi_{re}+2m_2\pi&=-k_2\sin\frac{2\pi}{5}\Delta x\end{aligned}. \tag{S18}$$

From equation (S18), we further obtain

$$4\cos^2\left(\frac{\pi}{5}\right)m_1=m_2-\frac{4\cos^2\left(\frac{\pi}{5}\right)-1}{2\pi}\varphi_{re}. \tag{S19}$$

After the integer point with coordinate (m_1,m_2) closest to the line with slope of $4\cos^2\left(\frac{\pi}{5}\right)$ and intercept of $\frac{4\cos^2\left(\frac{\pi}{5}\right)-1}{2\pi}\varphi_{re}$ is obtained, the 1D displacement is calculated as

$$\Delta x = -\frac{2m_1\pi + \varphi_{re}}{k_1 \sin\frac{\pi}{5}}. \tag{S20}$$

If φ_{re}=0.5π, the integer parameters were calculated as m_1=1 and m_2=3, and the 1D displacement is calculated was Δx=−1.1μm. As shown in Fig. S8 (a) and (d), S_z of the spin texture for N=5 applied with the phases (φ_1, φ_2, φ_3, φ_4, φ_5)=(0, 0, 0, 0, 0.5π) are the same as the one with displacements of Δx=−1.1μm and Δy=0μm. If the phases were changed to (φ_1, φ_2, φ_3, φ_4, φ_5)=(0, 0, 0, 0, 0.57π), the integer parameters were obtained as m_1=−4 and m_2=−10, and the 1D displacement was calculated as Δx=3.29μm, with the corresponding results shown in Fig. S8 (b) and (e). If φ_{re}=0.64π, the integer parameters were obtained as m_1=4 and m_2=11, and the 1D displacement was calculated as Δx=−3.83μm, with the corresponding results shown in Fig. S8 (c) and (f). It is found that changing the phase applied on the evanescent wave results in a discontinuous translation of the spin texture.

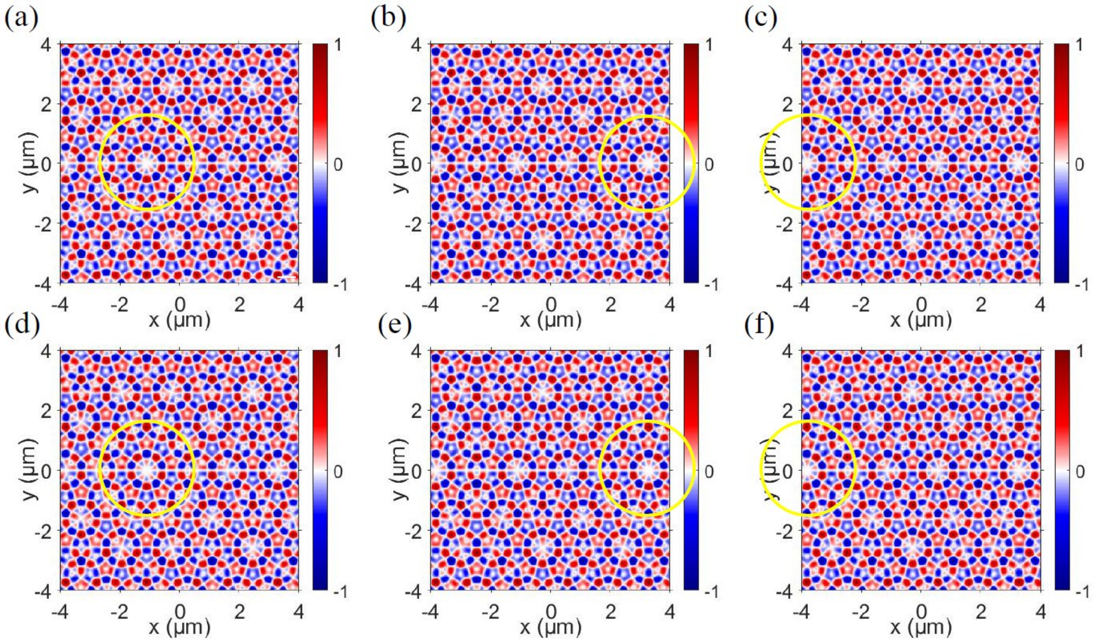

Fig. S8: 1D discontinuous translation of the photonic spin quasicrystals. (a–c) The longitudinal component of SAM with N=5 and applied phases (φ_1, φ_2, φ_3, φ_4, φ_5)=(0, 0, 0, 0, φ_{re}) with (a) φ_{re}=0.5π, (b) φ_{re}=0.57π, and (c) φ_{re}=0.64π. (d–f) The corresponding spin textures with the displacements of (d) Δx=−1.1μm, Δy=0μm, (e) Δx=3.29μm, Δy=0μm, and (f) Δx=−3.83μm, Δy=0μm.

Session 6: Displacement of the periodic spin texture for *N*=3

In the context of odd values of N, N=3 stands out as an exceptional case, for which the corresponding spin texture is periodic. According to equation (S11), we have

$$\begin{aligned}\varphi_1 &= k_1 \sin\frac{\pi}{3}\Delta x - k_1 \cos\frac{\pi}{3}\Delta y + \varphi_{re} \\ \varphi_2 &= k_1 \sin\frac{\pi}{3}\Delta x + k_1 \cos\frac{\pi}{3}\Delta y + \varphi_{re} \ . \\ \varphi_3 &= \varphi_{re}\end{aligned} \tag{S21}$$

For N=3, it is not necessary to introduce the integer parameters m_n to calculate the displacements. In this instance, the displacements are the analytical solutions of equation (S21) and are calculated as

$$\begin{aligned}\Delta x &= \frac{\varphi_1 + \varphi_2 - 2\varphi_{re}}{\sqrt{3}k_1} \\ \Delta y &= \frac{\varphi_2 - \varphi_1}{k_1}\end{aligned} \ . \tag{S22}$$

For example, the applied phases were set as (φ_1, φ_2, φ_3)=(π, 0.5π, 0), and the calculated displacements were Δx=0.153μm and Δy=−0.0885μm. As shown in Fig. S9, S_z of the spin texture for N=3 with applied phase is the same as the one with displacements.

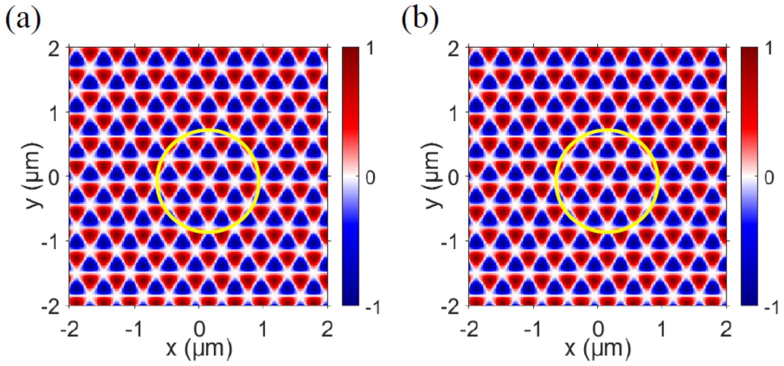

Fig. S9: Displacement of the periodic spin texture. (a) The longitudinal component of SAM with N=3 and applied phases (φ_1, φ_2, φ_3)=(π, 0.5π, 0). (b) The longitudinal component of SAM with N=3 and displacements of Δx=0.153μm, Δy=−0.0885μm.

Session 7: Experimental system for measuring the spin textures

The experimental set-up for generating and observing the quasi-periodic photonic spin texture is shown in Fig. S10. After passing through a telescope system, an incident laser beam with wavelength of 632.8nm illuminated the reflecting liquid SLM, which provided the phase profile Φ

$$\Phi = \arg\left[\sum_{n=1}^{N} e^{ik_t(x\cos\theta_n + y\sin\theta_n)} e^{i\varphi_n}\right], \tag{S23}$$

where k_t can be adjusted to match the entrance pupil of the objective. For example, the phase profile Φ for N=5 and the Fourier transform of $e^{i\Phi}$ are shown in Fig. S11 (a) and (b), respectively. It is found that the Fourier transform of $e^{i\Phi}$ manifests as N spots which are evenly distributed on a ring with the radius controlled by k_t. In the experiment, the modulated incident field was weakly focused by a lens with focal length of 500*mm* to perform the Fourier transform. The choice of focal length of the lens after the SLM was primarily based on two considerations. Firstly, a longer focal length lens is preferred to achieve light that closely resembles collimated light. Secondly, the choice of focal length can help to align the radius of the ring where the light spots are distributed. The N evenly distributed light spots were generated at the focal point, and then were focused by a 4*f* system onto the back focal plane of the objective. A combination of a linear polarizer (LP) and a m=1 vortex wave plate (VWP) was employed to turn the incident field into radial polarized. The incident beam was tightly focused by an oil-immersion objective (Olympus, NA=1.49, 100×) to excite the SPPs at the air/gold interface of the sample with 50-nm-thick gold film deposited on a glass substrate. The back focal image of the reflected beam from the sample was captured by the CCD and is shown in Fig. S11 (c). By adjusting k_t, the N evenly distributed light spots matched the dark ring which indicated the excitation of the SPPs. In this instance, the N evanescent waves were generated at the air/gold interface.

A polystyrene (PS) nanoparticle with a radius of 160 nm was immobilized on the gold film by the 4-mercaptobenzoic acid (4-MBA) molecular linker to scatter the transverse component of the near-field SPPs to the far-field for collection. The scattering radiation was collected by an objective (Olympus, NA=0.7, 60×), and the right and left circular polarized components of the scattering radiation were filtered out by a quarter-wave plate (QWP) and a LP and measured by the photo-multiplier tube (PMT, Hamamatsu R12829). The nanoparticle-on-film sample was fixed on a Piezo scanning stage (Physik Instrumente, P-545) to perform the two-dimensional scanning, and the two circular polarized components of the scattering radiation can respectively be mapped. The near-field distribution of the longitudinal SAM component can be expressed as (*44*)

$$S_z = \frac{\varepsilon}{4\omega i}\frac{k_r^2}{k_z^2}\left(E_x^* E_y - E_y^* E_x\right) = \frac{\varepsilon}{4\omega}\frac{k_r^2}{k_z^2}\left(I_{RCP} - I_{LCP}\right), \tag{S24}$$

where I_{RCP} and I_{LCP} indicate the right and left circularly polarized components of the in-plane electric field distribution, respectively. Therefore, S_z can be measured by simply measuring I_{RCP} and I_{LCP}.

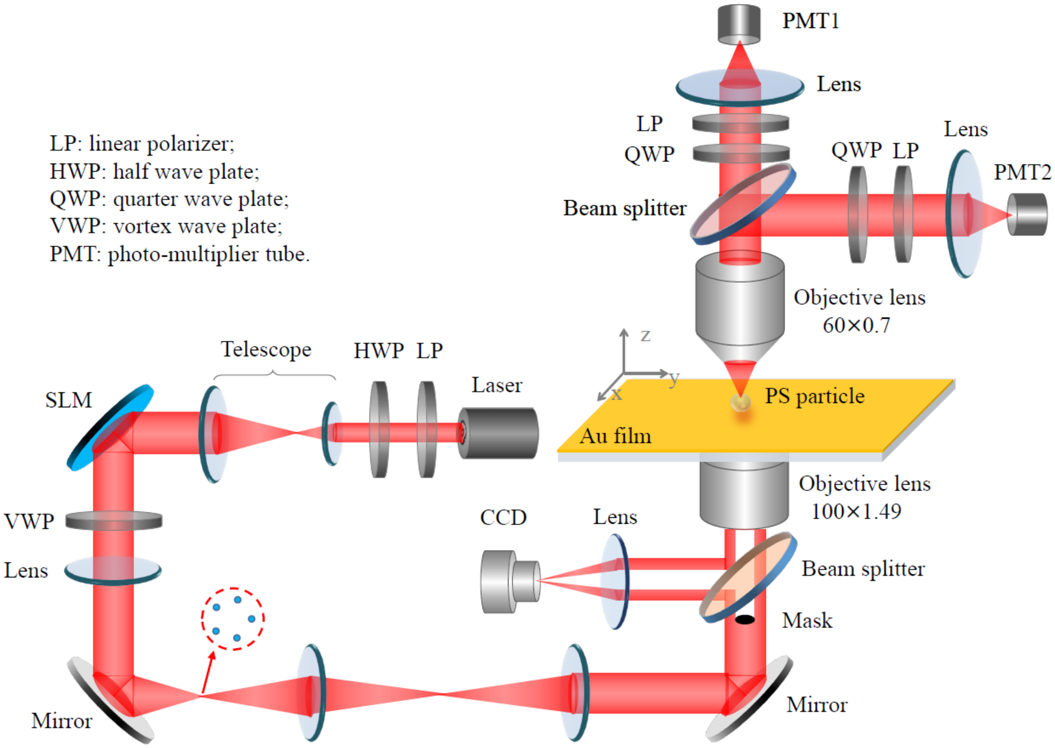

Fig. S10: The schematics of experimental set-up for measuring the spin texture.

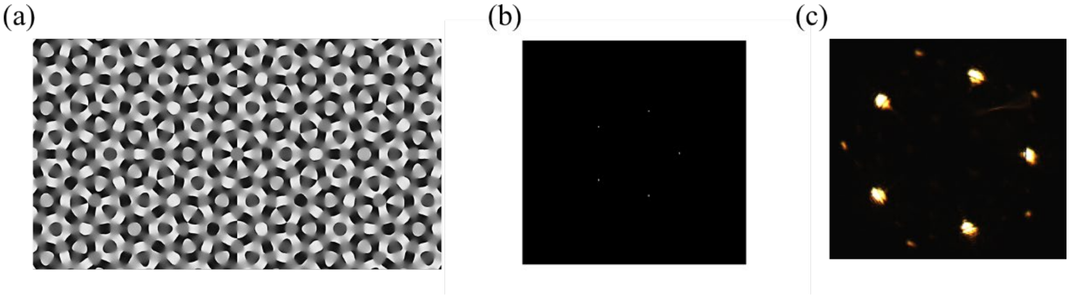

Fig. S11: The phase profile generated by the SLM. (a) The phase profile Φ for N=5. (b) The Fourier transform of $e^{i\Phi}$. (c) The back focal image of the reflected beam from the sample captured by the CCD.
